# Teleosts as behaviour test models for social stress

**DOI:** 10.3389/fnbeh.2023.1205175

**Published:** 2023-09-07

**Authors:** Nicola Hong Yun Lai, Izzati Adriana Mohd Zahir, Anthony Kin Yip Liew, Satoshi Ogawa, Ishwar Parhar, Tomoko Soga

**Affiliations:** Jeffrey Cheah School of Medicine and Health Sciences, Monash University Malaysia, Bandar Sunway, Selangor, Malaysia

**Keywords:** social stress, teleosts, emotion, social behaviour, cognition

## Abstract

Stress is an important aspect of our everyday life and exposure to it is an unavoidable occurrence. In humans, this can come in the form of social stress or physical stress from an injury. Studies in animal models have helped researchers to understand the body’s adaptive response to stress in human. Notably, the use of behavioural tests in animal models plays a pivotal role in understanding the neural, endocrine and behavioural changes induced by social stress. Under socially stressed conditions, behavioural parameters are often measured physiological and molecular parameters as changes in behaviour are direct responses to stress and are easily assessed by behavioural tests. Throughout the past few decades, the rodent model has been used as a well-established animal model for stress and behavioural changes. Recently, more attention has been drawn towards using fish as an animal model. Common fish models such as zebrafish, medaka, and African cichlids have the advantage of a higher rate of reproduction, easier handling techniques, sociability and most importantly, share evolutionary conserved genetic make-up, neural circuitry, neuropeptide molecular structure and function with mammalian species. In fact, some fish species exhibit a clear diurnal or seasonal rhythmicity in their stress response, similar to humans, as opposed to rodents. Various social stress models have been established in fish including but not limited to chronic social defeat stress, social stress avoidance, and social stress-related decision-making. The huge variety of behavioural patterns in teleost also aids in the study of more behavioural phenotypes than the mammalian species. In this review, we focus on the use of fish models as alternative models to study the effects of stress on different types of behaviours. Finally, fish behavioural tests against the typical mammalian model-based behavioural test are compared and discussed for their viability.

## 1. Introduction

Social stress which can be broadly defined as an emotional pressure or distress that arises from any social situations that threaten one’s self-esteem is a major source of stress for humans (Buwalda et al., 2005; Juth and Dickerson, 2013). Social stress can arise from daily interactions with family members, friends, and colleagues or from major life events such as losing a loved one or from an abusive relationship. For example, social isolation and social discrimination are forms of social stress that typically affect older aged people and the minorities in society. Social stress alters the neuronal structure, brain circuits and neurochemical composition, which then lead to changes in cognition, emotion, as well as social behaviour (Buwalda et al., 2005; Smeets et al., 2009). Understanding the underlying mechanism of stress response and stress-related diseases is essential to develop treatments and interventions to counteract the adverse effects of social stress and reduce the prevalence of stress-related diseases.

The use of animal models has greatly helped researchers to understand the effects of social stress on humans (Blanchard et al., 2001; Buwalda et al., 2011). Animal models are especially useful for social stress-related behavioural output studies. In a social stress study, behavioural parameters are vital components of focus, together with physiological and molecular parameters, for a complete understanding of the effect of social stress. This is because genetical, neurochemical or cellular alterations observed under social stress will eventually lead to changes in different types of behaviour including learning and memory, social behaviour, and aggression. Animal models can mimic the behavioural response of humans as well as the complex interaction between different parts of the brain under socially stressed conditions. Behavioural response of animal models can easily be assessed using various behavioural tests and the results are relevant to human as the social behaviour of vertebrates is controlled by the highly conserved social behaviour network (Goodson, 2005; Soares et al., 2018). Therefore, behavioural tests used in conjunction with different molecular tests in animal models is essential to narrow the gaps between how neural and endocrinological changes affect behaviour.

In recent years, the usage of fish models in the field of neuroscience has received more attention. Various experimental fish models and transgenic fish lines have been developed and used in different neuroscience research areas such as behavioural neuroscience, social neuroscience, and neurological disorders (Levin and Cerutti, 2009; Oliveira, 2013; Saleem and Kannan, 2018; Ogawa et al., 2021). The use of fish models in social stress research is also increasing that various social stress models such as social defeat stress (Higuchi et al., 2019; Lim et al., 2020), social isolation stress (Shams et al., 2017), and intruder/predator stress (Fischer et al., 2014; Xu et al., 2019) have been developed.

The teleost class accounts for 30,000 fish species, making up 98% of all ray-finned species. They boast impressive diversity in terms of morphology, physiology, and behaviour (Volff, 2005; Ravi and Venkatesh, 2018). On top of that, this group of fishes are highly social creatures, displaying both positive and negative social behaviours. In fact, the fish social brain network display a highly conserved profile of behavioural, neuroanatomical, hormonal, and molecular features as with other vertebrates (Ogawa et al., 2021). For instance, dopaminergic and noradrenergic neurons in medaka (*Oryzias latipes*) and zebrafish (*Danio rerio*) are found to have similar neuroanatomical networks and physiological roles with humans (Matsui, 2017). This makes the teleost fishes an attractive model for social stress studies. Many social stress studies have employed the zebrafish as their model, however, various other teleosts have the potential to be used in these studies, such as the African cichlids – *Astatotilapia burtoni*, Nile tilapia (*Oreochromis niloticus*), and Mozambique tilapia (*Oreochromis mossambicus*) – as well as the medaka, and the goldfish (*Carassius auratus*), amongst many other species. Furthermore, fish has a higher rate of reproduction, larger number of offspring at a time, and easier handling techniques as compared to mammalian models, easing the process of designing and conducting a study. The huge variety of teleost species provides flexibility in choosing appropriate fish models to investigate specific parameters or characteristics. For instance, highly socialized fish such as cichlid and salmonid fish are most suitable to be used as a model for social stress study (Gilmour et al., 2005; Félix and Oliveira, 2021). An advantage of fish models in social stress study as compared to typical rodent models is that the stress response in fish is found to be similar to humans where fish also secrete cortisol when stressed (Barton, 2002). Furthermore, the hypothalamic–pituitary-interrenal (HPI) axis in fish is homologous to hypothalamic–pituitary–adrenal (HPA) axis in humans (Winberg et al., 1997). In addition, some fish species exhibit diurnal or seasonal rhythmicity in response to stress, similar to humans, as opposed to rodents (Koch et al., 2017; Sánchez-Vázquez et al., 2019).

Many social stress studies have focused on molecular and physiological effects and mechanisms. However, these molecular mechanisms can also translate to different behavioural phenotypes, as a form of adaptation to stress or as a harmful, exacerbated response. Here, we will first discuss different types of social stress models available teleost, mainly zebrafish, as well as outline the various behavioural tests to measure the behavioural outputs that can be seen and observed due to social stress and molecular changes.

## 2. Social stress in teleost models

Teleost fish are highly social species. Thus, social stress set-ups have taken advantage of the various social behaviour observed in this model. At the moment, majority of the studies have been performed in zebrafish, with a review by Kareklas et al. highlighting its potentials as a model for social neuroendocrinology (Kareklas et al., 2023). As such, various social stress-inducing set-ups were made for zebrafish, although these set-ups can be adapted for other teleost species.

An important aspect of several teleost fish species is the ability to form social hierarchies within the population, resulting in dominant and subordinate fish. This particular trait causes infighting and social aggression between fish, mainly in males, in order to secure territory, nesting grounds, food, mates and breeding opportunities. Females may also become dominant and subordinate according to species or as part of parental behaviour, i.e., during mouthbrooding. Dominant males continuously act aggressively against subordinate males to maintain dominancy and territory. As a result of the lack of opportunities and resources, subordinates often have suppressed growth and reproductive function.

Social defeat paradigms are used to study the effects of antagonism by a more dominant conspecific or to observe the differences between dominant-subordinate fish. Various studies simply pair two fish of the same sex together, or form a small mixed sex group of fish, allowing them to interact and develop a social hierarchy (Larson et al., 2006; Filby et al., 2010; Dahlbom et al., 2011; Pavlidis et al., 2011; Tea et al., 2019; Bozi et al., 2021; Culbert et al., 2023). Dominant or subordinate fish are distinguished by their behaviour. Typically, dominant display far more aggressive behaviour, shown by chasing or biting behaviour towards subordinates, are often bigger in size, and in some species, may have a brighter body colouration, as observed in the Nile tilapia and *Astatotilapia burtoni* (Lim et al., 2020; Culbert et al., 2023). Dominants are less likely to show anxiety-like behaviour once they have obtained dominancy, and occupies a large territory, often in the middle of the tank. In contrast, subordinates display anxiety-like behaviour in their swimming. They are more likely to be swimming at the periphery of a tank, stay at the bottom, and display freezing behaviour. Subordinates are more likely to spend time fleeing from dominants and showcase less aggressive behaviours altogether. Many of these studies observed that a social hierarchy is formed early after initial pairing and the hierarchy is preserved for up to 5 days with no changes to social rank (Larson et al., 2006; Filby et al., 2010; Dahlbom et al., 2011; Pavlidis et al., 2011; Bozi et al., 2021; Culbert et al., 2023). This meant that once dominancy or subordinacy is established, is it less likely that the social rank will change after, particularly in experimental settings.

Another form of social defeat is by using previously established dominant fish. In our lab, we have studied the molecular effects of social antagonism on subordinates. This is done by placing a subordinate male taken from the housing tank into a tank with a bigger, dominant male. This ensures that the subordinate is continuously antagonized by a dominant, thus effects of subordinacy and social stress are more pronounced. We have performed two social defeat protocols, covering both acute and chronic social stress on the model species Nile tilapia (Higuchi et al., 2019; Lim et al., 2020; Thomas et al., 2021; Lim et al., 2023).

Being socially isolated has been linked to a plethora of problems with regards to social skills and health. In fish, a good social environment will lead to the development of social skills necessary for survival, such as predator inspection, foraging, and mate selection. Some species also showcase parental behaviour, with either only the mother, or father, or both parents participating in protecting and nurturing their young. Isolation studies simply require the separation of an individual from the shoal (Bai et al., 2016; Fulcher et al., 2017; Shams et al., 2017; Saszik and Smith, 2018; Tunbak et al., 2020; Marchetto et al., 2021; Shams et al., 2021; Onarheim et al., 2022). Several key points have to be taken into consideration when performing isolation studies. The age of separation depends on observing the effects of development isolation or isolating as an adult. Some studies separated fertilized eggs into their own tanks for the fish to develop and grow, only testing the effects at juvenile or adulthood (Bai et al., 2016; Fulcher et al., 2017; Tunbak et al., 2020; Shams et al., 2021). This addresses developmental isolation. Other studies separate adult fish from their shoals into their own containers (Shams et al., 2017, 2021; Saszik and Smith, 2018; Marchetto et al., 2021; Onarheim et al., 2022). Many studies ensure that the tank/beaker/petri dish has opaque walls to prevent any visual cues from the outside environment.

Researchers have also studied the effects of overcrowding. Overcrowding presents the challenges of lack of resources and space for food and nesting, increase of infighting, and overstimulation of social interactions. Overcrowding studies tests the effects of different tank densities (Ramsay et al., 2006; Gronquist and Berges, 2013; Saszik and Smith, 2018; Hou et al., 2019; Fontana et al., 2021c; Shishis et al., 2022; Tveit et al., 2022). Many studies of overcrowding focuses on fish welfare, particularly in long-term holding and aquaculture (Gronquist and Berges, 2013; Hou et al., 2019; Shishis et al., 2022; Tveit et al., 2022).

Another way to induce social stress employed by researchers is social instability. This paradigm creates an unstable social environment for the fish in a form of a disorganized social hierarchy (Moretz et al., 2007; Almeida et al., 2014; Border et al., 2021). Although there are limited studies employing this strategies, a few tactics have been published and used. Almeida et al. switched dominant male Mozambique tilapia between different tanks with established social environments for 7 days straight (Almeida et al., 2014). This creates an unstable environment where a shoal loses a dominant, creating space for a subordinate to ascend rank, while also introducing a new previously-dominant male. In contrast, the study had control tanks with stable environments, only picking out dominants then putting them back in without swapping them out to account for net handling stress (Almeida et al., 2014). Alternatively, Border et al. used male *Astatotilapia burtoni* and induced social instability with the removal of territory (Border et al., 2021). In the study, mixed sex shoals were placed in tanks with a number of flowerpots that may act as nesting grounds. Thus, dominant males will claim territory and defend its own flowerpot. The procedure included the removal and changing of the flower pots, simulating loss of territory, affecting the social ranks within the shoals (Border et al., 2021). Similarly, in another study by Moretz et al., different strains of juvenile zebrafish were mixed and grown together, to observe for the effects on the fish’s behaviour at adulthood (Moretz et al., 2007). This can act as a form of social stress and instability, as the different strains display different behaviours.

## 3. Behavioural tests in fish models

Social stress is unavoidable; thus, it is important to investigate the behavioural changes associated with social stress, in order to intervene accordingly. Social stress affects behaviour in different ways. Table 1 shows behavioural results of social stress studies. In the following sections, we divide the animal behaviour into three main categories for discussion which are emotion, social behaviours, and cognition. How specific tests can be applied to study social stress are discussed in the following sections.

**Table 1:** Summary of the findings from behavioural tests performed with different social stress paradigms in teleost models.

Behavioural test	Model	Social stress	Findings	Reference
Novel tank test	Adult, Mixed Sex Zebrafish	Social defeat	Increase in anxiety-like behaviour	Bozi et al. (2021)
	Adult, Male Zebrafish	Social defeat	No change in behaviour	Nakajo et al. (2020)
	Juvenile, Mixed Sex Zebrafish	Social defeat	No change in behaviour	Menezes et al. (2020)
	AB, Adult, Mixed Sex Zebrafish	Isolation	Decrease in thigmotaxic behaviour	Shams et al. (2017)
	Adult, Mixed Sex Zebrafish	Isolation	No change in behaviour	Onarheim et al. (2022)
Shoaling test	Juvenile, Mixed Sex Zebrafish	Social defeat	Increase in anxiety-like behaviour	Menezes et al. (2020)
	WT Short fin, Adult, Mixed Sex Zebrafish	Isolation	Decrease in social behaviour	Saszik and Smith (2018)
	WT Short fin, Adult, Mixed Sex Zebrafish	Overcrowding	Decrease in social behaviour	Saszik and Smith (2018)
	TM1 and Nadia strains, Juvenile and Adult, Mixed Sex Zebrafish	Social instability	Decrease in social behaviour	Moretz et al. (2007)
Mirror-biting test	Juvenile, Mixed Sex Zebrafish	Social defeat	Decrease in aggressive behaviour	Menezes et al. (2020)
	TM1 and Nadia strains, Juvenile and Adult, Mixed Sex Zebrafish	Social instability	Increase in aggressive behaviour	Moretz et al. (2007)
Social preference test	AB, Juvenile, Mixed Sex Zebrafish	Isolation	Decrease in social behaviour	Tunbak et al. (2020)
	TM1 and Nadia strains, Juvenile and Adult, Mixed Sex Zebrafish	Social instability	Decrease in social behaviour	Moretz et al. (2007)
T-maze test	Juvenile, *Cichlasoma paranaense*, unidentified sex	Isolation	Decrease in learning ability	Brandão et al. (2015)
Novel recognition test	Adult, Male Mozambique tilapia	Isolation	Decrease exploratory behaviour, increase in neophobia	Galhardo et al. (2012)

### 3.1. Emotions

In humans, exposure to bullying or workplace toxicity often leads to the development of depression and anxiety (Sittichai and Smith, 2015; Brouwers et al., 2016). However, the emotional impact of social stress is not only limited to humans but could also be replicated in rodent and fish models (Henriques-Alves and Queiroz, 2016; Bozi et al., 2021). Long-term exposure to social isolation or social defeat may lead to the development of depressive-like behaviours, such as helplessness and anhedonia (Chaouloff, 2013; Riga et al., 2015; Lei et al., 2022), as well as the development of anxiety-like behaviours (Ieraci et al., 2016; Ma et al., 2022). Quantification and assessment of depressive-like behaviours in fish poses a greater challenge than their mammalian counterparts. In mammalian models, measurement of depression via testing of anhedonia or despair can be established easily in rodents through the sucrose preference test, tail suspension test and the forced swimming test. In fish, however, behavioural phenotypes are often used as a substitute to determine depressive behaviours. Currently, a large number of tests conducted only measure anxiety-like behaviours, with limited emphasis on anhedonia and despair-like behaviours. General anxiety-like behaviours associated with fish includes freezing, defecation, hiding, as well as thigmotaxic behaviours (preference to walls when exploring an open spaces; Pellow et al., 1985; Stewart et al., 2012). However, more specific tests have been established to better understand and identify stress-induced behavioural phenotypes.

#### 3.1.1. Novel tank test

The novel tank test (NTT) is a test developed based on the open-field test used in rodent models to assess anxiety-like behaviour and locomotor activity. The procedure for both rodent and fish model are similar in which the animal is introduced to a novel open field environment and allowed to explore for a set period of time. For fish models, behavioural variables such as incidence of freezing, total distance travelled during test, swimming velocity and thigmotaxis (time spent near the wall vs. the center) are measured during the duration of the experiment (Godwin et al., 2012). The increased time spent in near the walls of the tank are indicative of anxiety-like behaviour. Other experiments also take into consideration the geotaxis (initial preference for the lower regions of the tank) behaviour of the fish (Bencan et al., 2009; Blaser and Rosemberg, 2012), whereby the duration spent at the bottom of the tank also corresponds to the degree of anxiety (Fontana et al., 2022). Several other factors could affect the results of NTT. For example, the direction of illumination of the novel tank, age of the subject fish, size of novel tank and the intensity of illuminating light are all factors to consider during the experiment (Haghani et al., 2019). As such, set-up of the experiment is an important aspect to consider prior to conducting the novel tank test. The thigmotaxis behaviours measured using this test have also been seen in humans to a certain extent (Gromer et al., 2021).

The use of NTT as a behavioural model to study anxiety-like behaviours has been tested on multiple species of teleosts, primarily in zebrafish (Godwin et al., 2012; Stewart et al., 2012), medaka (Matsunaga and Watanabe, 2010; Lucon-Xiccato et al., 2020, 2022), jundiá (Abreu et al., 2016) and guppies (*Poecilia reticulata*; Burns, 2008). However, some of these studies did not focus on the emotional aspect of the experiment and primarily investigated the exploratory behaviours, habituation, and locomotor activities in response to a novel environment. The NTT has been one of the main methods of investigation used to study anxiety-like behaviours in several social stress experiments involving teleost (Shams et al., 2015; Nakajo et al., 2020; Bozi et al., 2021; Onarheim et al., 2022). Anxiety behaviour after exposure to different types of social stress yielded conflicting results. Stress caused by social hierarchy were found to be apparent, as subordinate zebrafishes were found to display higher anxiety-like behaviour compared to their control and dominant counterparts (Bozi et al., 2021). On the other hand, Nakajo and colleagues reported that repeated exposure to social defeat had no significant impact on thigmotaxic behaviours in zebrafish as shown by the similar time spent in the upper portions of the tank between control and socially defeated zebrafish (Nakajo et al., 2020). Additionally, social isolation was initially thought to induce an obvious decrease in anxiety-induced thigmotaxic behaviours (Shams et al., 2015). However, Onarheim et al. (2022) observed that social isolation had no significant effect on zebrafish when subjected to NTT. It is important to recognize that results from both teleost and rodents highlight the inconsistencies of using OFT as a measuring tool for anxiety-like behaviours. Presumably, the use of such an over-simplified apparatus could give us an initial insight to changes in behavioural phenotype. However, other tests must be complimented together with the OFT to further understand the full behavioural effects of stress exposure.

#### 3.1.2. Scototaxis test

Another commonly used tool to identify anxiety-like behaviours in fish is the light–dark preference/scototaxis test. The procedure involved placing the fish into a half-black, half white tank for habituation. Then, the fish is allowed to freely explore the tank without any intervention. The number and duration of entries in each compartment are recorded by the observer for the whole session (Maximino et al., 2010). Increased activity in the white compartment is indicative of anxiolytic behaviours, whereas increased activity in the dark compartment is indicative of anxiogenic-like behaviours (Maximino et al., 2010). In addition to the compartmental activity stated above, additional parameters such as frequency of body and caudal fin (BCF) transient propulsions (Webb, 1984), frequency of ‘freezing’ events, vertical distribution (top versus bottom of the water column; Levin et al., 2007; Echevarria et al., 2008), ventilation rate (Barreto and Volpato, 2004), and changes in the coloration of the animal (Gerlai et al., 2000; Hoglund et al., 2000; Maximino et al., 2010) can also be additional parameters used to evaluate anxiety-like behaviours. The light/dark test has been mostly tested in zebrafish (Steenbergen et al., 2012; Holcombe et al., 2013; Magno et al., 2015; Baiamonte et al., 2016). However, other species of fish such as the bluegill (*Lepomis macrochirus*; Yoshida et al., 2005), crucian carp (*Carassius langsdorfi*; Yoshida et al., 2005), goldfish (Gouveia et al., 2005; Yoshida et al., 2005; Maximino et al., 2007), guppy (Maximino et al., 2007), Nile tilapia (Maximino et al., 2007), lambari (*Astyanax altiparanae*), cardinal tetra (*Paracheirodon axelrodi*; Maximino et al., 2007), banded knifefish (*Gymnotus carapo*; Maximino et al., 2007), blacksmith damselfish (*Chromis punctipinnis*; Kwan et al., 2017), onesided livebearer (*Jenynsia multidentate*; Calcagno et al., 2016), black perch (*Embiotoca jacksoni*; Hamilton et al., 2014) and the three-spined stickleback (*Gasterosteus aculeatus*; Porseryd et al., 2019) have also reported similar scototaxic behaviours.

The main advantage of the light/dark test is that this test presents a clear conflict situation for the fish in which the fish need to decide between staying in preferred protected areas (e.g., black compartment) and an innate motivation to explore novel environments. Under normal physiological conditions, fish tend to prefer darker compartments as compared to the bright departments (Maximino et al., 2010). Treatment of fish models with anxiolytic drugs led to increase exploration and time spent in the light compartment as well increase locomotion (dos Santos Sampaio et al., 2018; Costa de Melo et al., 2019; Lachowicz et al., 2021). Hence, we believe that application of the light/dark preference test to fish models is a reliable method to examine anxiolytic drugs.

However, it is still unclear whether the scototaxic behaviours of fish is due to the actual presence of anxiety-like behaviours or due to other potential factors. For example, the scototaxic behaviours of medaka, who are subjected to intense predation in the wild (Nakao and Kitagawa, 2015), were found to be significantly different than zebrafish, whereby the medaka spent equal amounts of time in the light and dark compartments (Lucon-Xiccato et al., 2022). Hence, the scototaxis test cannot be blindly implemented into any experimental design and requires a better understanding of the nature of the fish before subjecting them to any experimental paradigms.

#### 3.1.3. Elevated plus maze

The elevated plus maze test is another widely used behavioural assay to assess anxiety-like behaviours in rodents (Walf and Frye, 2007) and several attempts have been made to mimic the EPM test in fish, primarily in zebrafish. Amongst them, the swimming plus maze test designed by Varga and colleagues, is one of the first attempt to replicate the experimental design of the EPM (Varga et al., 2018). Similar to EPM in rodents, their experimental setup involves a “+” shaped platform consisting of 2 + 2 opposite arms, different in depth, connected by a center zone. A more detailed protocol is explained by Varga et al. (2018). In this experiment, both larval and juvenile zebrafish were found to have increased activity in deeper arms compared to shallow arms (Varga et al., 2018). Presumably, these bottom dwelling behaviours are similar to the thigmotaxis behaviour displayed in rodents, with an increased time being spent in the bottom being an indication of anxiety-like behaviours (Blaser and Goldsteinholm, 2012). In this experiment, administration of anxiolytic compounds, such as buspirone and chlordiazepoxide, caused an increase in shallow arm activity whereas administration of an anxiogenic compound, such as caffeine, promoted deep arm preference and activity (Varga et al., 2018). Taken altogether, the swimming plus maze test functions as an alternate method of measuring anxiety, utilizing the zebrafish’s unique characteristic of preferring greater depths over shallower surfaces (Blaser and Goldsteinholm, 2012; Blaser and Rosemberg, 2012). Another variation of the EPM test is the submerged plus maze test developed by Hope et al. (2019). Compared to Varga’s version (Lai et al., 2020) of the maze test, which is specific to the zebrafish’s preference for deeper surfaces, this version relies on the scototaxic behaviour of the fish. The submerged plus maze apparatus is shaped as a plus symbol with alternating black (black fill) and transparent (white fill, dashed lines) arms, this results in two visually closed arms, two visually open arms and a center area. Behaviour scores are then given based on the number of lines crossed per area, amount of time spent in each area, total number of lines crossed, number of entries into new arms as well as number of entries into open arms (Hope et al., 2019). Higher anxiety-like behaviours are categorized by higher activity in the black arms, while higher activity in the transparent arms is indicative of a lack of anxiety. Both aquatic adaptations of EPM appear similar at first, but they measure anxiety based on different behavioural phenotypes. Although both may seem adequate for screening of anxiolytic drugs, their use in social stress studies is questionable and has yet to be proven. Regardless, future studies should attempt to incorporate both thigmotaxis and scototaxis behaviours when developing future versions of the EPM in fish models.

#### 3.1.4. Zebrafish tail immobilization test

One of the main consequences of social stress is the emergence of despair-like behaviours. In humans, this is can be seen through the portrayal of learned helplessness, which refers to the failure of individuals to pursue, utilize, or acquire adaptive instrumental responses (Nuvvula, 2016). In typical rodent models, assessment of learned helplessness can be observed either through the tail suspension test (TST) or the forced swimming test. The study of behavioural despair in teleost models has recently garnered attention. Indeed, several studies have noted the ability of zebrafish to display helpless responses akin to their mammalian counterparts (Lee et al., 2010; Andalman et al., 2019; Mu et al., 2019). However, behavioural experiments to investigate and assess behavioural despair is difficult to implement in fish models. Analogous to the tail suspension test (TST) in rodents, the ZTI is the first known method of measuring behavioural despair in fish (Demin et al., 2020a). In TST conducted in rodents, the animals are suspended midair by their tails, which will then develop into an immobile posture (Porsolt et al., 2001; Cryan et al., 2005). The lack of escape-related behaviours or activities is indicative of despair-like behaviour and is measured via total duration of immobility and number of immobility episodes (Ueno et al., 2022). In ZTI, this experiment involves the vertical suspension and the immobilization of the caudal half of the body while leaving the cranial part to move freely in a small beaker of water. In contrast to the TST, the ZTI test measures the total ‘activity’ of the zebrafish when immobilized instead of total immobility time, with ‘activity’ being defined by the total distance moved during the experiment by the center-point of the free handing zebrafish’s body (Demin et al., 2020a). Despair-like behaviour is then characterized by reduced ‘activity’ which mimics the learned helplessness state experienced by rodents. Theoretically, the ZTI could be used to study learned helplessness and despair-like behaviours in social stress models. However, this has yet to be proven. The ZTI is the first to show and measure despair-like behaviours in teleost. Application of the ZTI is currently limited to zebrafish, however its potential implementation in other species of fish seems promising. One potential issue is that there is currently a lack of additional studies to verify the validity of this behavioural experiment. The current interpretation of results is primarily based on our current understanding of behavioural despair in rodent models, but how our understanding of behavioural despair in rodents may not exactly translate to in fish models. Furthermore, implementation of the TST in other species of fish could prove challenging in bigger sized fish and could require additional set-up.

#### 3.1.5. Anhedonia

Another symptom of social-stress induced depression is the presence of anhedonia. Anhedonia is defined as the loss of interest or pleasure in daily activities, which is a core symptom of depression (Moreau, 2022). In rats, chronic stress was found to decrease reward-seeking behaviours, exploration, and locomotor activity in rodents, and these reductions are regarded as loss of motivation. Anhedonia has been extensively studied in zebrafish with a recent review highlighting the behavioural tests used to study the various phenotypes of anhedonia (de Abreu et al., 2022). Amongst them, the conditioned place preference (CPP) test could be implemented to mimic the effects of the sucrose preference test in rodents. The sucrose preference test is a reward-based test which is commonly used to measure anhedonia. A reduction in the sucrose preference ratio in experimental relative to control rodents in indication of anhedonia (Root et al., 2018). In zebrafish, the CPP could theoretically be used to measure social-stress induced anhedonia in fish. In the CPP paradigm, zebrafish showed preference for the environment that has previously been associated with a substance (typically a drug), hence indicating the positive-reinforcing qualities of that substance (Mathur et al., 2011). Anhedonic behaviours can then be evaluated by the lack of preference to the drug-associated environment. Furthermore, assessing anhedonic behaviours could also be conducted via several social tests, which are discussed below. Currently, most studies have primarily focused on the development of anxiety-related behavioural assays. In hindsight, the most optimal behavioural paradigm to achieve this is through the aquatic adaptations of the EPM, which could potentially incorporate the thigmotaxic, geotaxic and scototaxic behaviours into its design. Besides that, a myriad of studies has also been conducted to evaluate anhedonic behaviours in zebrafish. However, it’s incorporation to other fish models may not be applicable due to the difference in species. One of the current limitations of using fish to study symptoms of depression is the lack of behavioural paradigms investigating learned helplessness (behavioural despair). Hence, there is a need to expand upon this field for future studies involving depression.

### 3.2. Sociability

The act of being social is fundamental and inherent to any living being, conserved across species, between humans and primates, to rodents and fish. Humans live in communities and form meaningful and supportive connections with each other. In fact, stable and supportive social interactions lead to longer life expectancies in humans (Holt-Lunstad et al., 2010). The social brain network is an evolutionary conserved structure in the vertebrate brain (Sandi and Haller, 2015; Ogawa et al., 2021). Likewise, the social brain network of fish is homologous to that of mammals (Ogawa et al., 2021). Stressful experiences from conspecifics can prompt changes in the social brain network at the level of functionality, neurochemical, epigenetic mechanisms, and structural changes, resulting in behavioural changes (reviewed by Sandi and Haller, 2015). Thus, observing for changes in social behaviours can indicate dysfunction in the social brain network and be a good tool for stress studies. Early life stress experiences are linked to increased aggression, social anxiety, and isolation in adult humans (Sandi and Haller, 2015). Victims of childhood bullying had high involvement with drug abuse and crime at a later age (Lee et al., 2022). Studies in rodents have also found altered social behaviours after exposure to stress. Early social isolation of mice decreased social behaviour and interest to a conspecific (Kuniishi et al., 2022). Social instability in adolescent female rats reduced social interactions (Asgari et al., 2021). Thus, it is clear that stress linked to social factors can impact social behaviour. Fish showcase a vast array of social behaviours (Ogawa et al., 2021). For example, fish can display social decision making in response to their social environment and interactions. Teleosts like the Nile tilapia and *Astatotilapia burtoni* have social hierarchies that involve dominant and subordinate males. These positions are not fixed and can change according to the situation; subordinate males are quick to assert dominance once the previous dominant male is no longer present. A few behavioural tests have been developed to study social behaviour in fish. Majority of studies were conducted on zebrafish and were used to reliably evaluate the effects of toxins and pollutants that can affect behaviour. Therefore, these tests can also be used to show social behavioural changes in social stress studies. The behavioural tests may also be altered for different fish species accordingly.

#### 3.2.1. Mirror-biting test

The mirror-biting test is used to study aggression in fish (Pham et al., 2012; Way et al., 2016; Audira et al., 2018). Heightened aggressive behaviour is often seen as a response to stress and may be detrimental for social interactions. However, even aggression can be seen as normal social behaviour due to the tendency for teleosts to form social hierarchies. Therefore, even a lack of aggression can be seen as impacted social behaviour. The set-up is simple, where a tank with a mirror attached on one of its walls is used. Several points of measures are taken, including the time taken to first approach, time taken to first contact, number of approaches and contact. Contact is seen as “butting” or “biting” the fish’s own reflection on the mirror. Pusceddu et al. (2022) explained that the test can be performed in two ways with slight differences, (1) putting in a mirror into an occupied tank, or (2) adding in the test fish into a tank with an already attached mirror. The first method may trigger the zebrafish’s defences of an ‘intruder’ into its space, while the second method tests novelty towards both the mirror and tank. The zebrafish will first display wariness to the mirror and gradually build to approach. The “biting” behaviour will likely occur early on during the test but as the time goes on, the fish will start to get used to its reflection. Heightened aggression is characterized by higher number of approaches and ‘biting’ behaviour. An alternative method to evaluate aggression in fish is by pairing up with a real opponent conspecific and staging a ‘fight’. This is a simple method where the test fish are tagged and placed together in the same tank, with their interactions recorded for observation, similar to the resident-intruder paradigm in rodents (Teles and Oliveira, 2016). Often, a dominant and a subordinate will be established by the end of the pairing. The same fish can be paired up with other fish to observe for their ‘fighting’ behaviour as according to their ‘winner’ or ‘loser’ status. Indeed, ‘winners’ display more aggressive behaviour than that of the ‘losers’ (Oliveira et al., 2016).

Juvenile zebrafish that faced aggressive, alcohol-influenced male adults displayed low levels of aggression towards their own reflections (Menezes et al., 2020). Stressed adult male zebrafish, in the form of ‘losers’ in staged fights, are likely to lose their subsequent fights, indicating lower motivation, while the ‘winner’ fish are more likely to win their next fights (Oliveira et al., 2011; Nakajo et al., 2020). Thus, it can be seen that social stress can reduce aggression, induce fear and avoidance behaviour, with lower social motivation in stressed zebrafish. This meant that these zebrafish are less likely to assume dominant positions in the shoal, thus, reducing mating opportunities and access to food and resources. The impact of social subordination on reducing aggression seems to be conserved between juvenile and adult zebrafish. In contrast, social instability stress from being grouped with a different zebrafish strain induced an increase in aggressive behaviour compared to pure-strain groups (Moretz et al., 2007). Thus, exposure to other behaviours from other strains can impact aggressive behaviour.

#### 3.2.2. Social preference test

The social preference test can be used to measure the ability to recognize and interact with conspecifics. In animals with no social deficits, it is likely that there is a preference towards their conspecifics to be social rather than being alone. In fact, anhedonia-like behaviour induced by stress can be observed through the social preference test. Social anhedonia is the loss of pleasure in social interactions and lower social motivations, seen in debilitating conditions such as major depressive disorder and schizophrenia (Enneking et al., 2019; Cohen et al., 2020).

A few protocols have been published for zebrafish, with slight differences (Pham et al., 2012; Audira et al., 2018; Norton et al., 2019), and is comparable to the rodent three-chamber sociability test (Kaidanovich-Beilin et al., 2011; Rein et al., 2020). Essentially, a large tank is used and divided into smaller chambers of the same size, separated by removable, transparent barriers. According to Pusceddu et al. (2022), these chambers can be designated as conspecific chamber, conspecific arm, middle chamber, empty arm, and empty chamber. Conspecific fish, as a shoal or just a singular fish, act as social stimulus and are placed into the conspecific chamber. The other end is designated as the empty arm. There are several ways to measure sociability from this test, including, counting the number of entries into either conspecific arm or empty arm, comparing the time spent in a specific arm with the total test time, or a ratio of time in the conspecific arm versus the empty arm. Higher sociability is shown by spending more time in the conspecific arm. This behaviour may be affected in fish with social deficits. To make it even simpler, a three-chamber apparatus can be used, with only a conspecific arm, a holding chamber in between, and an empty chamber. Swimming close to either chamber indicates preference. Norton and colleagues modified the social preference protocol slightly to test for social novelty (Norton et al., 2019). The first phase consists of a similar social preference set-up mentioned above, but a second phase is added to test curiosity and interaction towards a novel conspecific or shoal. An unfamiliar group of conspecifics is added to a different chamber. The two variables of interest include the time spent near the familiar conspecific chamber and the time spent near the novel chamber. Typically, the zebrafish should spend more time exploring the novel, unfamiliar shoal, indicating higher social behaviour.

Ogi and colleagues published a systematic review detailing zebrafish studies using the social preference test (Ogi et al., 2021). Some criteria may need to be considered when designing the social preference test for a study. Preference towards a shoal seemed to be a learned criterion, where zebrafish preferred the same species conspecifics or is similar in phenotype (Engeszer et al., 2004; Saverino and Gerlai, 2008). At the same time, zebrafish tend to prefer bigger conspecifics compared to smaller ones (Aslanzadeh et al., 2019). A few studies have employed the social preference test to evaluate for behavioural changes after social stress. Male adult zebrafish that faced repeated social defeat did not display any changes to social preference towards conspecifics, including lower aggression (Nakajo et al., 2020). In contrast, isolated juvenile zebrafish displayed significantly lower social behaviour by spending lesser time near its conspecifics, and showcased long durations of freezing behaviour (Tunbak et al., 2020). Additionally, an unstable social environment in juvenile zebrafish lead to lower social behaviour and moving away from shoal stimulus, although this effect was strain specific (Moretz et al., 2007). Thus, it is likely that the different forms of social stress is able to induce different reactions to the zebrafish’s social preferences. Outside of social stress, acute physical stress reduced the zebrafish’s preference to its conspecifics (Giacomini et al., 2016). Similarly, acute stress on the jundiá fish made them less social, as evaluated from the lower number of entries and time spent in the zone near conspecifics (Abreu et al., 2016). Interestingly, untreated zebrafish reacted negatively to pain in the social preference test. Untreated fish preferred buffer treated zebrafish over acetic acid-treated fish, likely due to visual pain manifestations (Rosa et al., 2022). This shows that the social preference test can be used to study the fish’s reaction to distress and test its perception in other conspecifics. A possible alternative for the social preference test set-up includes using transparent tanks and lining them up together, thus, possibly accommodating bigger fish and reduce costs on getting special equipment.

#### 3.2.3. Shoaling test

The shoaling test is used to evaluate overall social behaviour and is specific to fish (Pham et al., 2012; Way et al., 2016; Audira et al., 2018). Zebrafish, and many other fish species, naturally form shoals which represent complex interactions in coordinated movements. In fact, shoaling is thought to be protective and an anti-predator behaviour as being in a group reduces risk of being caught (Paijmans et al., 2020). Shoaling may act as a ‘social buffering’ mechanism, reducing the effects of stress on individual fish and hasten recovery. Anhedonic-like behaviour can also be potentially observed in this test, together with a reduction in social motivation.

The set-up simply requires a group of zebrafish in a tank. A line can be drawn horizontally across the tank to separate the top and bottom parts. With a shoal, several endpoints can be taken. Generally, the spread of the group can indicate levels of anxiety. A tighter, closely packed shoal is likely to be more anxious, while a loose shoal indicates lower levels of anxiety. Other parameters such as inter-distance between closest neighbours, average inter-distance between individuals of the shoal, or percentage of fish at the top half of the tank versus the bottom half (for more details see Pusceddu et al., 2022).

Exposure to an aggressive adult resulted in juvenile zebrafish to form tighter shoals with low average interindividual distances compared to naïve, unexposed controls (Menezes et al., 2020). Both isolation and overcrowding resulted in loose cohesion within adult zebrafish shoals, with observations of greater distance between neighbours, indicating lower sociability after these two forms of social stress (Saszik and Smith, 2018). As such, different social stresses, in this case, aggression versus isolation and crowding, will cause different responses in shoaling. The opposing results may also be due to the age of exposure to social stress. Zebrafish formed tighter shoals in response to a new environment, but loosened up after a period of time (Kleinhappel et al., 2019), indicating that social interaction increased after an acute stressor, likely to reduce risk of danger. Indeed, being in a group has positive and calming effects on fish, including, reducing metabolic demands in the damselfish (*Chromis viridis*; Nadler et al., 2016), increase swimming efficiency in the thicklip grey mullet (*Chelon labrosus*; Hemelrijk et al., 2015), and better decision-making effects against predators in the mosquitofish (*Gambusia holbrooki*; Ward et al., 2011). Additionally, chronic unpredictable stress had caused anxiety-like behaviour and tighter shoals than controls (Demin et al., 2020b). Therefore, tighter shoals indicate a stress response behaviour and adaptation against stress and predator.

### 3.3. Cognitive function

Cognition can be defined as the ability to acquire, retain, and use information or knowledge (Kihlstrom, 2018). This includes learning, memory formation, attention and using the information to coordinate motor outputs. Under socially stress condition, the affected emotion will in turn influence cognitive functions including attention, working memory, and motivational or reward-based decision making (Okon-Singer et al., 2015). A recent longitudinal study in the United States found social stress is associated with declining episodic memory and executive functions (Lindert et al., 2022). Cognition can be tested using behavioural tests as one’s cognitive ability can be presented through their behaviour. The fish species is proven to have cognitive ability where the fish is able to learn and form memory (Brown, 2020; Salena et al., 2021). Several studies even reported numerical cognition in fish (Agrillo et al., 2017; Vila Pouca and Brown, 2017). However, only few social stress studies using fish models have included cognitive functions as a parameter. Since it is known that social stress can affect cognitive functions, cognitive behavioural tests should be performed in social stress study for drug discovery or neural and molecular mechanism for a comprehensive understanding of social stress effects. Common behavioural tests used in fish models to assess the fish’s cognitive ability are condition place preference/avoidance, Y-maze, T-maze, and novel object recognition test.

#### 3.3.1. Conditioned place preference/conditioned place avoidance

Conditioned place preference/aversion (CPP/CPA) is a behavioural paradigm based mainly on the principles of classical conditioning (Tzschentke, 2010) used to assess memory consolidation and retention ability (Blank et al., 2009). Social stress studies use more of the CPA paradigm, also known as the inhibitory avoidance test, than the CPP. The assay includes using a tank with two distinct compartments separated by different visual cues. The visual cues can be bright/dark, clear/dot or colored/uncolored. A pre-test can always be carried out to determine if the animal prefers any of the compartment. For CPA procedure, the animals will first be placed in the unpreferred compartment and given a punishment upon entry into the preferred compartment. The number of training sessions can vary depending on study design (Blank et al., 2009; Manuel et al., 2014; Lai et al., 2020). The test session includes measuring the latency to enter the compartment paired with aversive stimulus. Memory intact animals will show increased latency to enter the compartment after training, indicating that they recall the punishment associated with entry. Meanwhile for the CPP paradigm, a reward is paired with the unpreferred compartment, and the time spent in the previously unpreferred compartment measured. The time taken to enter the opposite compartment during training and test sessions are usually recorded (Bertoncello et al., 2022). Difference between the latencies from training to test session can be calculated as index of memory retention. Due to the nature of CPP/CPA assay that associate decision with punishment or reward, the effect of social stress on reward-oriented decision-making could also be evaluated.

Although no study has yet to use the test in fish models for social stress study, the test has been used in rodent models. Chronic social defeat stress in mice is found to cause a decrease in inhibitory avoidance behaviour (Monleón et al., 2015). The finding indicates that social stress may impact memory formation and the reward-seeking cognitive functions resulting in less reward-seeking behaviour or impaired memory consolidation. On the other hand, in other type of stress study, Tuebingen long-fin zebrafish were found to have decreased inhibitory avoidance learning and memory formation ability after chronic unpredictable stress (Manuel et al., 2014). Interestingly, the study found that the results were more profound in the zebrafish group exposed to stress during resting phase, compared to during the active phase. For example, not only did the fish stressed at night had higher whole-body cortisol levels, the gene expression levels of corticoid receptor genes and *bdnf* were increased, indicating higher responses to cortisol and higher neuronal differentiation. These changes in molecular level may be the factors influencing the learning and memory of zebrafish under stress conditions. In addition, goldfish is also shown to have inhibitory avoidance behaviour (Faganello et al., 2003; Faganello and Mattioli, 2007; Faganello and Mattioli, 2008). Thus, CPP/CPA assays are suitable behaviour-specific tests to study rewards-based decision making or memory formation under social stress in teleost fish models.

#### 3.3.2. Y-maze test

The Y-maze test, mainly used to assess spatial memory, is named after the apparatus used in the test which is a Y-shaped tank with arms of identical length. Different protocols have been utilized in Y-maze. The commonly used tests are novel arm recognition test (Cognato et al., 2012; Pecio et al., 2022) and spontaneous alternation test (Fontana et al., 2021a,c). The novel arm recognition test usually includes two sessions. The animal model is allowed to explore the tank in the first session, however, one arm is closed off. Next, during the test session, the animal will be allowed to freely explore all three arms. The time spent in each arm, total distance traveled, mean speed, and turn angle are all recorded. This test is developed based on the fact that the animal naturally prefers exploring novel environments instead of familiar environments. Spending more time in the novel arm indicates the animal memorized which of the arms it had previously explored, reflecting an intact spatial memory. Geometrical cues can also be given outside the tank or stuck on the wall of the arm to help the animal recognize different arms. Another application of Y-maze is the spontaneous alternation or also known as free movement pattern (FMP) Y-maze test in fish models. The test is simple in which the animal is placed into the Y-maze tank and allowed to freely explore all three arms (Fontana et al., 2021a,c). The turning choice of the animal is recorded. A correct alternation is determined by continuously visiting a new arm and not previously visited arm. Recognizing a novel arm is an indication of spatial learning and memory ability. The test can be carried out with geometrical cues outside the maze or no extra cue is given. The work done by Cleal et al. further demonstrate that the FMP Y-maze can be translated into humans for assessing working memory (Cleal et al., 2021).

Fish have spatial memory and often use spatial reference for foraging in nature (Brown, 2020). Therefore, fish models are suitable to be used for the study of spatial and working memory. Y-maze has been carried out in zebrafish (Cognato et al., 2012), goldfish (Ohnishi, 1997), *Astyanax fasciatus* (Burt de Perera and Holbrook, 2012), and Arctic char (*Salvelinus alpinus*; Håkan Olsén and Winberg, 1996). Although no work has related social stress and Y-maze test in fish but other type of stress studies has been carried out. Studies in zebrafish have demonstrated chronic unpredictable early-life stress (CUELS) only affects the FMP Y-maze performance of adult zebrafish when stress is given to fish of 1 week old but not 6-weeks-old (Fontana et al., 2021b,c). The findings suggest that spontaneous alternation in Y-maze test is sensitive to the timing of stress exposure. Besides, research using rodent models has shown social stress is able to impair Y-maze performance. For example, chronic socially defeated rodents fail to recognize novel arm in Y-maze test (Tian et al., 2018; van der Kooij et al., 2018). Furthermore, aged mice subjected to chronic social stress during adolescence show impaired Y-maze performance (Sterlemann et al., 2010). Study in rats also show social-single prolonged stress reduced the spontaneous alternations in Y-maze (Fulco et al., 2022). Taken together, social stress can affect the performance of animals in the Y-maze test, thus, this test should also be included in social stress study to assess spatial and working memory.

#### 3.3.3. T-maze

T-maze is a popular behavioural test in rodent models for assessing working memory and has been adapted to be used in fish models. For fish models, T-maze is a four-day protocol with 3 days of learning following a test session on the fourth day mainly used to assess spatial learning and memory (Kaur et al., 2022). Generally, the apparatus is a T-shape tank with one long arm and two short arms. The short arms design could vary depending on the needs of the experiment. Some T-maze tanks come with deeper chambers in the short arms (Pusceddu et al., 2022), or the walls of the short arms are coded with color. Normally the fish will learn to associate one arm with reward or punishment. The fish can be given rewards (food or enriched environment; Braida et al., 2020; Kim et al., 2022) or punishment (mild electric shock or disturbance by glass rod; Kaur et al., 2022) when it enters a designated arm depending on the study need. Punishment is normally used to test for avoidance learning while rewards test for spatial learning and memory. The fish will undergo training for days to reinforce the learning of avoidance or entries. On the test day, parameters such as the latency to move, number of entries into correct arms, and latency to enter either arm are measured. Intact learning and memory ability are represented by correct entries of arm. Similar to CPA/CPP, T-maze is also dependent on emotion or motivation for completion of cognitive task. Therefore, T-maze is also a relevant cognition behaviour test for social stress to test reward-based decision making.

Social isolation in a cichlid fish, *Cichlasoma paranaense* is found to impair learning in T-maze (Brandão et al., 2015). On the other hand, in rodent models, both social defeat and chronically social stressed rats showed impaired performance in T-maze suggesting the test is sensitive to stress and suitable for assessing cognitive functions (Ferragud et al., 2010; Novick et al., 2013). In addition, the T-maze apparatus is also used in other teleost fish model including common carp (*Cyprinus carpio*; Garina et al., 2016), killifish (*Kryptolebias marmoratus*; Rossi and Wright, 1953), mangrove rivulus (*Kryptolebias marmoratus*; Chang et al., 2012), Port Jackson sharks (*Heterodontus portusjacksoni*; Byrnes et al., 2016), goldfish (Romaguera and Mattioli, 2008; Facciolo et al., 2011, 2012), grey bamboo shark (*Chiloscyllium griseum*; Schluessel and Bleckmann, 2012), ornate wrasses (*Thalassoma pavo*; Zizza et al., 2017), mosquitofish (Vinogradov et al., 2021), Siamese fighting fish (*Betta splendens*; Craft et al., 2003, 2007; Shapiro and Jensen, 2009), *Girardinus falcatus* (Bisazza et al., 2001), guppy (Miletto Petrazzini et al., 2017) for different purposes such as learning test, lateralization and decision-making study. T-maze is an apparatus with great flexibility for assessing cognitive functions and the performance is sensitive to stress exposure, thus, should be incorporated in future social stress study as one of the behavioural parameters.

#### 3.3.4. Novel object recognition test

Another popular cognitive test in fish models is the novel object recognition (NOR) test (DePasquale et al., 2021). This test includes three phases. Firstly, the fish is introduced to the experimental tank without anything for habituation. The time for the fish to acclimatize to the tank varies from minutes (May et al., 2016) to days (Kaur et al., 2022). Then, two similar objects are placed into the tank at equal distances from each other. The fish is reintroduced into the tank in the middle chamber and allowed to explore. After that, one of the objects is replaced with a novel object. The fish is introduced into the tank again after different time intervals and allowed to explore. The time spent around each object is recorded and presented as proportion of time spent around the object or proportion of total time. Intact memory formation ability is represented by spending more time around the novel object. This test exploits the fact that the fish prefer exploring a novel object (Gaspary et al., 2018).

However, there are a few points that need to be noted for this test. The personality of the fish will affect the results. For example, juvenile fish tend to be shyer, thus, might favor familiar objects more while bold fish will explore more around novel objects (Lucon-Xiccato and Dadda, 2014). Moreover, the size, pattern, and color of the object also influences the test results. A bigger object might be seen as a predator to the fish, and fish tend to have color preference (Kawamura et al., 2010; Roy et al., 2019). Hence, to minimize the effect of the influencing factors, a pre-test can be carried out using a non-experimental but the same batch of fish to ensure the fish do not have prior preference towards one of the objects used.

NOR has not been popularly used for social stress experiments in fish. However, this test has been used in zebrafish (DePasquale et al., 2021), guppy (Lucon-Xiccato and Dadda, 2016), *Astatotilapia burtoni* (Wallace and Hofmann, 2021), Caribbean bicolour damselfish (*Stegastes partitus*; Hamilton et al., 2017), and European chubs (*Squalius cephalus*; Santos et al., 2021), in other studies, showing that fish models are able to recognize novel and familiar objects. Besides, findings from rodents’ study suggested social stress impaired recognition memory especially in vulnerable individuals. For instance, the recognition index of social stress susceptible mice is significantly lower than the control or resilient mice (Huang et al., 2013; Duque et al., 2017; Costa et al., 2022). However, interestingly, social isolation in female rats did not affect the recognition memory as both control and stressed rats explored the object at novel location more (McCormick et al., 2010). In fact, both fish and rodents have reported sex differences in NOR probably due to sex differences in exploring behaviour (Lucon-Xiccato and Dadda, 2016; Bath et al., 2017; Agarwal et al., 2020; Wallace and Hofmann, 2021). In addition, social status of fish is found to be able to affect the NOR preferences where lower status fish prefer familiar object while dominant male fish prefer novel object (Wallace et al., 1845). Besides, in *Oreochromis mossambicus*, social isolation cause impaired NOR behaviour (Galhardo et al., 2012). Both findings collectively show that the social environment could affect NOR results.

Furthermore, fish are known to have social memory and can recognize conspecific, the test can be further modified into social discrimination test, a test used in rodent models for assessing social memory. Instead of object, conspecific is used to test if the animal will explore the novel or familiar animal more (Sterlemann et al., 2010). In fact, a similar test has already been employed in zebrafish model (Kumari et al., 2023). Social memory can be affected during social stress condition; Therefore, the test is useful to assess social cognition.

### 3.4. General discussion

Research has been carried out to understand the impact and/or neural and molecular mechanism of social stress using animal models for discovery of new treatment or drug target to counterpart the negative effects of social stress. Behavioural parameters present as a vital component in a study for understanding the consequences of stress or the effect of anxiolytic drugs. Emotion and sociability related behavioural tests are more commonly used for social stress study while cognitive related tests have been used less. This may be due to most of the study only focusing on emotion and social behaviour components under stress conditions. Nevertheless, cognitive functions are also closely related and influencing emotion and social behaviour, therefore, cognition related behavioural should also be included in social stress study. For example, a study using cognitive behavioural test found that monoamine oxidase inhibitor helps to protect the brain of goldfish from the effect of social defeat stress and prevent cognitive decline (Thangaleela et al., 2021). This study has demonstrated the application of cognitive behavioural tests together with molecular parameters in social stress study can help elucidate the mechanism of social stress effects. Furthermore, using multiple types of behavioural test in one study a more holistic understanding of the stress effects. For instance, using mirror-biting test and NTT, exposure to aggressive adult during juvenile phase in zebrafish shown to decrease aggressive behaviour later in adult phase without changing the anxiety level or locomotor activity (Menezes et al., 2020), suggesting previous stress experience might reduce the motivation for social interaction but does not cause anxiety-like behaviour. By using just two different types of tests, it may be ruled out that in the study the reduced aggression in adult zebrafish is not due to heighten anxiety level but may be caused by other factors.

In addition to the usage discussed above, most of the behavioural tests can be further modified to answer different research questions. For instance, Y-maze in fish models can also be modified to carry out barrier task same as in rodent models. Besides, T-maze can be used in different scenarios to assess different behaviour in fish such as memory and learning, despair-behaviour, and lateralization. Various parameters can be recorded and analyzed for a test such as latency to move, latency to enter correct arms, number of entries, time spent in correct arms, and mean speed. A video of the fish during the test is normally recorded and analyzed using different software. Besides, for the tests that include learning and trial phase, the fish can be categorized as learner or non-learner (Brandão et al., 2015), while short-term and long-term memory can be assessed with different time intervals between training phase and trial phase (Fu et al., 2021). Taken together, the behavioural tests are flexible to suit different social stress studies. Similar to rodent’s behavioural tests, the tests in teleost fish models are not perfect and hold limitations. Most of the tests mentioned above are widely used in zebrafish models only but application to other fish models is possible with a few considerations to note. Some pre-tests should be done to ensure the results are reliable and comparable. For example, fish have different light/dark preference, color preference (Roy et al., 2019), and preference for novel objects, thus, a pre-test is important to determine the fish preference to ensure these influencing factors have been considered while designing the test. It should also be noted that fish within the same species can have different learning ability. For instance, Tupfel Long-Fin zebrafish rapidly learns to avoid the dark compartment while AB strain zebrafish show no inhibitory avoidance learning (Gorissen et al., 2015). Furthermore, the results of each test are sensitive to external factors such as the presence of investigator and external cue, thus, the environment where the test is carried out should be kept constant for comparable and reproducible results. The results produced are affected by the motivational status of the fish as well because motivation/emotion affects the willingness of fish to swim (Wood et al., 2011). Moreover, the majority of the cognition related behavioural tests only assess spatial memory which is non-declarative memory. Although newer behavioural tests for declarative memory are developing in fish (Andersson et al., 2015), the progress is definitely slower than for rodent models. Moreover, emotion related behavioural tests are also limited to assessing anxiety-like behaviour while test for measuring despair-like or anhedonia behaviour is still scarce. Nevertheless, behavioural tests in fish are generally on smaller scales and less time consuming while producing highly reliable data that are comparable to rodent models.

## 4. Future perspectives

The similarity of social stress response between fish and humans brain makes fish model particularly relevant for translational research perspectives. We have discussed some evidences that fish behaviour studies hold a key role in understanding of brain functions and pathophysiology in various social stress associated neurological disorders in this review. There is a prospect in future of using fish as biomedical research model in investigating of brain dysfunctions linked behavioural changes, based on the advantages and current accomplishment of fish behavioural studies. We suggest some directions for future behavioural research in fish, particularly as it relates to social stress response, mental disorders as well as cognitive functions. The behaviour experimental approach must be defined according to the type of social stress and phenotype in model fish. There are some differences between mechanisms underlying variation in social stress responses in different species. Although zebrafish as a model is well-established in behavioural research areas as same as developmental biology, genetics, and pharmacology, we need to open our mind into other social fish to improve technical limitations and the variation of behavioural patterns during social stress. Laboratory environment might lead highly variable housing conditions and become a huge impact on the individual behavioural change, physiological variance and social stress response of fish. It is urgently necessary to set the standardization of these behavioural experimental approaches to achieve a quantitative understanding of fish behaviours during social stress. We also need to consider different levels of behaviour, based on perception and response of social stress in fish. As one of ongoing technological advances, machine learning may offer complementary data modelling techniques to those in current behavioural data analysis. Machine learning technology can address difficult tasks, such as classifying species, and individuals, or acquire, identify, and interpret behavioural patterns within complex fish behavioural data. Besides, it provides useful means to do so in ways that embrace its natural complexity. These new behavioural approaches using machine leaning offer the best means to advance this research field of social stress and social stress-related disorders.

## 5. Conclusion

Teleost presents as a robust model for social stress studies due to its various social behaviours and conserved social brain network to mammals. Research has focused on the molecular and physiological aspects of social stress responses; however, social stress may lead to different behavioural responses as well. Most social stress set-ups in fish exploit the natural social behaviours observed in the teleost, including the formations of social hierarchy in shoals resulting in dominant and subordinates, as well as the need of social interactions during development and adulthood. Different types of social stress models in fish, will impact on emotions, social behaviour, and cognition differently. Therefore, the behavioural tests above can be done alongside current molecular studies, allowing for more robust findings to the effects of social stress. Although limited, we have also made the observation that different social stressor at different ages can affect various behaviours. At the moment, zebrafish remains as the prime fish model. With adjustments, these behavioural tests may also be used in other teleost species, presenting a larger avenue for social stress studies.

## Author contributions

NL, IM and AL wrote the first draft. SO, IP, and TS edited the first draft. All authors contributed to the article and approved the submitted version.

## Conflict of interest

The authors declare that the research was conducted in the absence of any commercial or financial relationships that could be construed as a potential conflict of interest.

## Publisher’s note

All claims expressed in this article are solely those of the authors and do not necessarily represent those of their affiliated organizations, or those of the publisher, the editors and the reviewers. Any product that may be evaluated in this article, or claim that may be made by its manufacturer, is not guaranteed or endorsed by the publisher.
